# Degradation of Stains from Metal Surfaces Using a DBD Plasma Microreactor

**DOI:** 10.3390/mi15030297

**Published:** 2024-02-21

**Authors:** Fajun Wang, Zhikun Miao, Chengdong Li, Liangliang Lin

**Affiliations:** Key Laboratory of Synthetic and Biological Colloids, School of Chemical and Material Engineering, Ministry of Education, Jiangnan University, Wuxi 214122, China; 6220606072@stu.jiangnan.edu.cn (F.W.); 7230610015@stu.jiangnan.edu.cn (Z.M.); li690612564@jiangnan.edu.cn (C.L.)

**Keywords:** DBD plasma microreactor, microplasma, metal cleaning, plasma surface treatment

## Abstract

The surface cleaning of metals plays a pivotal role in ensuring their overall performance and functionality. Dielectric barrier discharge (DBD) plasma, due to its unique properties, has been considered to be a good alternative to traditional cleaning methods. The confinement of DBD plasma in microreactors brings additional benefits, including excellent stability at high pressures, enhanced density of reactive species, reduced safety risks, and less gas and energy consumption. In the present work, we demonstrated a DBD plasma-based method for the degradation of stains from metal surfaces in a microreactor. Aluminum plates with capsanthin stains were used to investigate the influence of operational parameters on the decolorization efficiency, including plasma discharge power, plasma processing time, and O_2_ content in the atmosphere. The results revealed that an increase in plasma discharge power and plasma processing time together with an appropriate amount of O_2_ in the atmosphere promote the degradation of capsanthin stains. The optimum processing condition was determined to be the following: plasma power of 11.3 W, processing time of 3 min, and Ar/O_2_ flow rate of 48/2 sccm. The evolution of composition, morphology, bonding configuration, and wettability of aluminum plates with capsanthin and lycopene stains before and after plasma treatment were systematically investigated, indicating DBD plasma can efficiently degrade stains from the surface of metals without damage. On this basis, the DBD plasma cleaning approach was extended to degrade rhodamine B and malachite green stains from different metals, suggesting it has good versatility. Our work provides a simple, efficient, and solvent-free approach for the surface cleaning of metals.

## 1. Introduction

Metals are essential in the development of human civilization and have become an indispensable part of modern society. To date, they have been extensively used in numerous fields, such as construction, electronics, automobiles, electronics, and aerospace, etc. Surface quality is an important criterion to evaluate the overall performance and functionality of metals as it is closely related to their key properties like mechanical strength, wear resistance, hardness, corrosion protection, and biocompatibility [1]. However, the surface contamination of metals is inevitable during practical applications, which may seriously affect their mechanical properties and functionality [2]. To maintain a high performance, the surfaces of metals need to be cleaned to remove undesired impurities.

Traditional cleaning approaches, based on chemical cleaning or mechanical cleaning, have been widely used to remove surface contaminants from metals, such as paint, rust, oil, and grease [3]. However, in some cases, organic solvents involved in chemical cleaning are undesirable. They may cause severe damage to metals or even destroy their functionality when used for long periods [4]. Furthermore, they also have negative impacts on human health and the environment. As to mechanical cleaning, it relies on direct actions on the surfaces of metals to remove contaminates. Compared to chemical cleaning, mechanical cleaning is simpler, cheaper, and more environmentally friendly, without using hazardous organic solvents [5]. Further enhancement can be achieved using machinery equipment or systems to increase cleaning efficiency, where a large surface area can be cleaned within a short time. On the other hand, limitations still exist in mechanical cleaning. For instance, it is commonly labor-intensive, time-consuming, and has poor security. The surfaces of metals might be damaged by mechanical forces, leading to reduced mechanical strength. With the increasing use and growing demand for high performance metals in industries, it is crucial to develop a green and efficient method for cleaning metals without damage.

Plasma has been regarded as a novel and green technique in chemistry and chemical engineering. It contains abundant reactive species like electrons, excited atoms, ionized gas molecules, and photons [6]. Once applied in surface cleaning, these radicals can rapidly break the chemical bonds of surface-attached contaminations. Currently, there are already studies on non-thermal plasmas for the removal of organic and inorganic chemical residues. For example, Vasikaran et al. [7] used an atmospheric air-assisted plasma reactor for the oxidative degradation of azo (orange G, Congo red) and non-azo (crystal violet, Coomassie brilliant blue) textile dyes. Korner et al. [8] demonstrated an Ar/H_2_ discharge for the removal of inorganic contaminations from metallic and silicon substrates. Krüger et al. [9] reported a plasma etching process to remove contaminants like air pollutants, fingerprints, oxide layers, or additives from the surface of different materials (e.g., glass, metals, carbon, and polymers).

As one of the most popular and classical plasmas, DBD plasma is characterized by the presence of at least an insulating material between electrodes [10,11]. It offers several key advantages over traditional cleaning methods, including simple operations, a green and efficient process, reduced surface damage, no organic solvents and hazardous disposals, etc. Furthermore, it can generate stable and uniform discharges on a large scale at atmospheric pressure and room temperature and is easily scalable from small laboratory reactors to large manufacturing setups [12,13]. It also produces a lot of reactive oxygen species (ROS) like H_2_O_2_, O_2_^−^, ·OH, HO_2_^−^, and O_3_, etc., which are very useful in the decomposition of contaminates from the surfaces of metals. These characteristics make DBD plasma very suitable for industrial cleaning. The integration of DBD plasma with microreactors to form DBD microplasma brings additional benefits. According to previous studies, plasmas confined within a sub-millimeter-length scale have an enhanced density of reactive species, a strong non-equilibrium state in a wide range of gas mixtures, and reduced safety risks while requiring less gas and energy consumption [14,15,16]. In addition, due to its strong non-equilibrium effects, electrons have sufficient energy to collide with the background gas to produce charged particles, metastables, fast neutrals, radicals, and photons, etc. These species largely enhance its effectiveness in cleaning metal surfaces. Based on these reasons, microreactors’ plasmas have been intensively researched in recent years. For instance, a fluidic rolling robot using voltage-driven oscillating liquid was proposed to realize the rolling motion [17]. A droplet generator with its power sources on a chip was fabricated to realize the generation of a droplet with a desirable diameter and production rate [18].

In this work, the surface cleaning of metals was researched using a DBD plasma microreactor, aiming to realize the green and efficient degradation of stains without damaging bulk metals. Operational parameters were explored to obtain the optimum processing condition. Al plates with capsanthin and lycopene stains were treated in the microreactor to investigate the evolution of samples before and after plasma treatment. The versatility of the DBD plasma cleaning technique was evaluated by extending the stains as well as metals to different materials.

## 2. Materials and Methods

### 2.1. Materials

Capsanthin (>97%) and lycopene (98%, HPLC) were purchased from Beijing Mairuida Technology Co., Ltd. (Beijing, China) Rhodamine B (AR) and malachite green (AR) were purchased from Sinopharm Group Chemical Reagent Co., Ltd. (Shanghai, China). Aluminum plates, copper plates, iron plates, and stainless-steel plates were provided by AnHui Zhengyang Mechanical Science and Technology Co., Ltd. (Lu’an, China). Argon (≥99.999%) and oxygen (≥99.999%) were supplied by Wuxi Taihu Gas Co., Ltd. (Wuxi, China).

### 2.2. Sample Preparation

**Figure 1:**
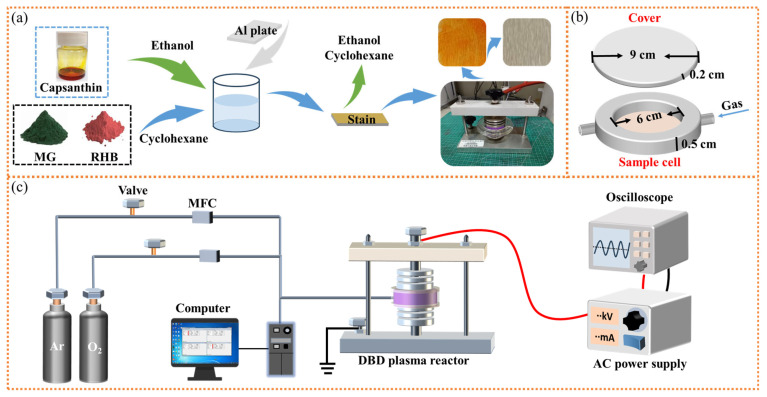
(**a**) Preparation of the metal plates with certain types of stains; (**b**) the quartz DBD plasma microreactor; (**c**) DBD plasma setup used in this work.

The sample preparation procedures were depicted in Figure 1a. Specifically, metal plates were carefully cleaned with ethanol and dried under ambient conditions. A certain amount of capsanthin was used as the stain and dissolved in cyclohexane to obtain the corresponding solution (1 wt%). RhB and MG were dissolved in ethanol to obtain the corresponding solutions (1 wt%). Then, the entire metal plates were immersed in the above solutions for 3 min, before being taken out to completely evaporate the cyclohexane. The as-prepared metal samples were coated with capsanthin, RhB, and MG stains. All samples were processed via the same procedure to ensure the maximum consistency among samples. For lycopene, it was uniformly distributed on the surfaces of metal plates to get the samples with stains.

### 2.3. DBD Plasma Setup

In the present work, experiments were carried out in a quartz DBD plasma microreactor, as shown in Figure 1b. The bottom part of the microreactor is a dish containing a sample cell (diameter = 6 cm, thickness = 0.5 cm), while the upper part is a flat quartz plate that exactly covers the dish (diameter = 9 cm, thickness = 0.2 cm). For each operation, the microreactor was placed between the electrodes of an atmospheric pressure DBD plasma setup (Figure 1c). One of the electrodes was connected to an AC high-voltage power supply (CTP-2000K, Nanjing Suman Electronics Co., Ltd., Nanjing, China), while the other electrode was grounded. Argon and oxygen were used as the working gas, and their flow rates were controlled by mass flow controllers (Bronkhorst High-Tech BV, Ruurlo, The Netherlands) in the pipeline. Prior to plasma processing, samples were placed in the microreactor. The reactor was then flushed by the working gas for 5 min to remove any possible impurities. With the supply of high voltage to the electrodes, plasma discharges were formed within the microreactor. Experimental data were recorded using an oscilloscope (Tektronix TBS1102, Beaverton, OR, USA). After a desired processing time, the plasma processing was switched off, and samples were carefully taken out for further characterization.

### 2.4. Characterization

Fourier transform infrared (FT-IR) spectra were recorded using a Nicolet 6700 infrared spectrometer (Thermo Fisher Scientific Inc., Waltham, MA, USA). Raman spectra were recorded using a Renishaw inVia Raman microscope (Renishaw, Gloucestershire, UK). UV-Vis diffuse reflection spectra were obtained using a UV-3600 plus spectrophotometer (Shimadzu, Tokyo, Japan). The morphology and topography of the samples were examined using a VHX-1000C super depth-of-field microscope (SDFM, Keyence, Hong Kong, China) and a Multimode 8 atomic force microscope (AFM, Bruker Daltonics, Billerica, MA, USA). The detailed morphology of samples was observed using an S-4800 scanning electron microscope (SEM, Hitachi, Tokyo, Japan), with a silicon drift energy dispersive X-ray spectrometer (EDX) to acquire elemental information. X-ray photoelectron spectroscopy (XPS) characterization was carried out using a Kratos Axis Ultra DLD spectrometer (Kratos, Manchester, UK) that was equipped with an Al Kα monochromatic X-ray source (1486.7 eV). The wettability of the samples was examined using an OCA 40 contact angle instrument (Eastern Datephy, Beijing, China).

## 3. Results

**Figure 2:**
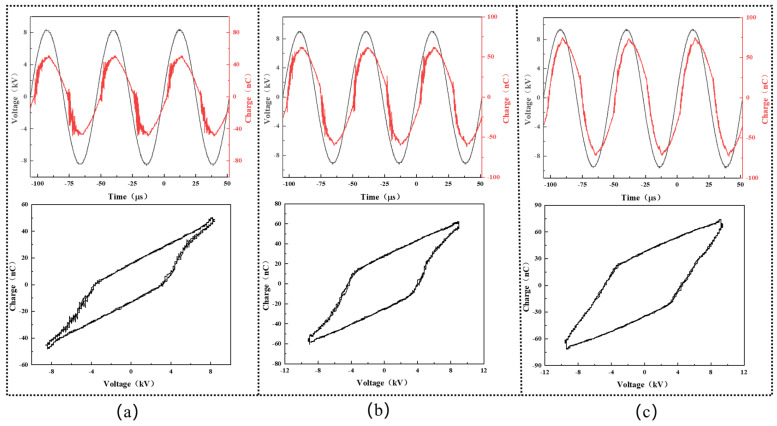
V-Q diagrams and the corresponding Lissajous curves during plasma degradation of stains at diverse plasma discharge powers: (**a**) plasma discharge power of 5.5 W; (**b**) plasma discharge power of 11.3 W; (**c**) plasma discharge power of 17.3 W.

Figure 2 shows the representative waveforms of the applied voltage and transported charge during the experiment. Periodic discharge events are observed in all cases, with nearly the same values of the voltage and charge in the positive half-cycle and the negative ones. In our study, the discharge power was varied to study its effect on the decolorization efficiency of the DBD plasma cleaning technique and calculated from the below Lissajous curve [19]:

(1) $$\bar{P}=\frac{1}{T}\oint V_{a}dQ_{m}$$

where $\bar{P}$ is the plasma discharge power, *T* is the AC cycle period, *V_a_* is the instantaneous voltage, and *Q_m_* is the instantaneous capacitor charge. On this basis, the plasma discharge power was calculated to be 5.5 W, 11.3 W, and 17.3 W, as seen in Figure 2a–c, respectively.

The decolorization efficiency *Dr* (%) was evaluated against aluminum plates with the capsanthin stain at diverse plasma powers using the whiteness measurement:

(2) $$Dr=\frac{R_{1}-R_{2}}{R_{3}-R_{2}}$$

where *R*_1_ and *R*_2_ are the whiteness values of the tested sample after and before the plasma processing. *R*_3_ is the whiteness value of the original sample.

**Figure 3:**
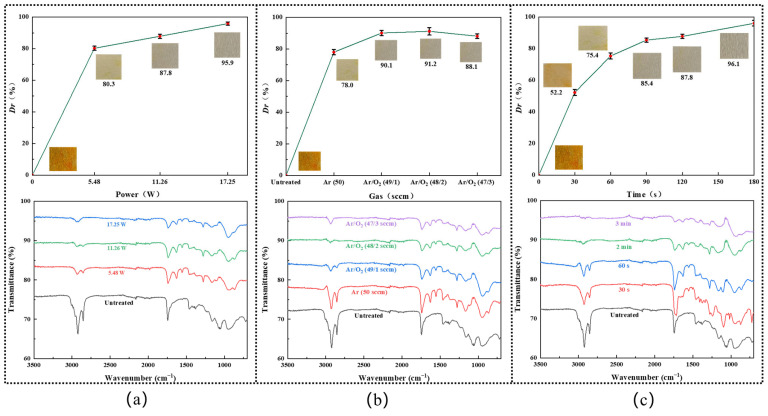
Decolorization efficiency and FT-IR spectra of the capsanthin treated with plasma at different conditions: (**a**) different plasma discharge power, plasma processing time: 2 min, plasma atmosphere: Ar/O_2_ (48/2 sccm); (**b**) different atmosphere, plasma discharge power: 11.3 W, plasma processing time: 2 min; (**c**) different plasma processing time, plasma discharge power: 11.3 W, plasma atmosphere: Ar/O_2_ (48/2 sccm).

Figure 3a shows the decolorization efficiency of the DBD plasma at diverse plasma powers, with Ar/O_2_ (48/2 sccm) atmosphere and 2 min processing time. One can see the decolorization effect was drastically increased with the rise of the plasma discharge power, from nearly 80.2% at 5.5 W to 87.6% at 11.3 W, and finally reached around 95.6% at 17.3 W. FT-IR spectra were recorded to study the functional groups of the samples before and after plasma treatment. Characteristic peaks relating to the capsanthin molecule were observed in all samples without plasma processing. Specifically, the strong absorption peak at 3010 cm^−1^ is attributed to the stretching vibration of the =C–H bond. The absorption peaks at 2925 cm^−1^ and 2852 cm^−1^ are due to the stretching vibration of the methylene and methyl C–H bonds, respectively. The peak at 1745 cm^−1^ is indexed to the C=O stretching [20]. Less intensive peaks at 1465 cm^−1^ and 1375 cm^−1^ are originated from the planar rocking and bending vibrations of the methylene and methyl C–H bonds. However, with the rise of the plasma discharge power from 5.5 W to 17.3 W, the intensities of the capsanthin-related absorption peaks gradually weakened. At a plasma discharge power of 17.3 W, the C=O absorption peaks were significantly reduced, and the C–H absorption peaks disappeared. This suggests the effective degradation of capsanthin. Considering that large plasma power lacks competition in terms of economic efficiency, the plasma discharge power was fixed at 11.3 W. On the other hand, new absorption peaks are observed in the range of 900–1000 cm^−1^, which are attributed to the generation of oxygen-containing groups. Among them, the most apparent one located at around 950 cm^−1^ is assigned to O–H bending and should be caused by the dissociation of water vapor to introduce hydroxyl groups on metal surfaces [21].

The influence of the O_2_ content on the decolorization efficiency of capsanthin was studied via the whiteness measurement and FT-IR spectra (Figure 3b). One can see the decolorization efficiency initially improved with the rise of the O_2_ content in the range of 0–2 sccm and then reduced when the O_2_ flow rate exceeded 2 sccm. A maximum decolorization efficiency was achieved in Ar/O_2_ (48 sccm/2 sccm) atmosphere (Figure 3b). This is because the addition of O_2_ in plasma would lead to the formation of reactive oxygen species (ROS) like H_2_O_2_, O_2_^−^, ·OH, HO_2_^−^, and O_3_, etc. They can react with the capsanthin molecules to dissociate them rapidly. Meanwhile, the density of the ROS increased with the rise of the O_2_ flow rate, resulting in enhanced decolorization efficiency. On the other hand, it should be noted that the production efficiency of ROS in Ar/O_2_ DBD plasma will be reduced once the oxygen concentration exceeds a certain value [22]. Thus, subsequent experiments were carried out in Ar/O_2_ (48/2 sccm) atmosphere.

By operating the plasma at 11.3 W in Ar/O_2_ (48/2 sccm) atmosphere, while varying the plasma processing time in the range of 0–3 min, the influence of plasma duration on the decolorization efficiency of capsanthin was investigated. One can see the decolorization efficiency gradually increased with the prolonged plasma treatment time, from 52.6% at 30 s to 76.0% at 60 s, 85.1% at 90 s, and 87.6% at 120 s. Once the plasma treatment time reached 3 min, the capsaicin color almost completely disappeared, with a decolorization efficiency of 95.89% (Figure 3c). Such a phenomenon was also reflected by the FT-IR spectra, where the intensities of the absorption peaks of capsanthin drastically reduced.

**Figure 4:**
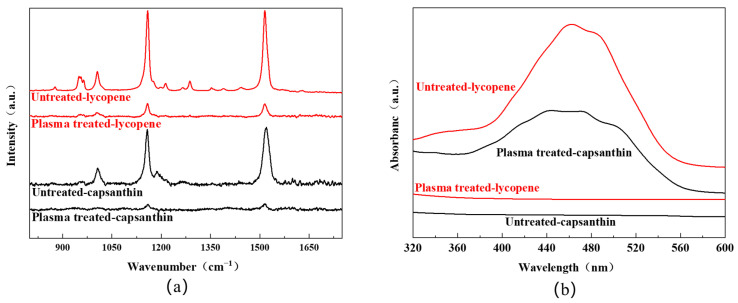
(**a**) Raman spectra and (**b**) UV-Vis diffuse reflection spectra of the aluminum plates with lycopene and capsanthin before and after plasma treatment.

Figure 4a shows the Raman spectra of the aluminum plates with stains like lycopene and capsanthin before and after plasma treatment. All experiments were carried out at a plasma power of 11.3 W, with a plasma processing time of 3 min and an Ar/O_2_ flow rate of 48/2 sccm. For the sample with lycopene, characteristic Raman peaks of lycopene were observed. Specifically, the peak at 964 cm^−1^ was attributed to the bending vibration of the C=C bond. The peak at 1005 cm^−1^ was due to the antisymmetric telescopic vibration and bending vibration of the methyl group. The distinct peaks at 1154 cm^−1^ and 1521 cm^−1^ were assigned to the telescopic vibration of the C=C and C–C bond [23,24]. After plasma treatment, the intensity of the Raman peaks greatly reduced, suggesting that lycopene was effectively degraded. For the sample with capsanthin, Raman peaks were detected at 1522 cm^−1^, 1155 cm^−1^, and 1004 cm^−1^, corresponding to the stretching vibration of the C=C bond, the vibration of the conjugated polyene chain, and the bending vibration of methyl groups in capsanthin, respectively. These peaks disappeared after plasma processing, indicating the degradation of capsanthin.

In addition to the Raman analysis, UV-Vis diffuse reflection spectra were recorded to examine the samples with/without plasma treatment. One can see that all samples before plasma processing display a broad band in the range of 400–550 nm, with the maxima at around 460 nm (Figure 4b). This band is originated from the conjugated polyene structure of the molecules [25]. After plasma processing, the absorption band completely disappeared, indicating the destruction of the conjugated polyene structures. The result is in agreement with the whiteness measurement, FT-IR, and Raman spectra. Therefore, DBD plasma can serve as an efficient way for the degradation of stains from metal surfaces.

**Figure 5:**
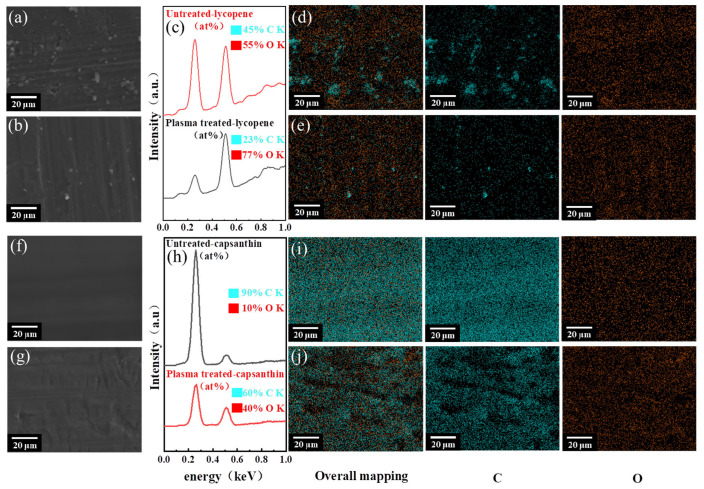
SEM images, EDX spectra, and elemental mapping of Al plates with lycopene and capsanthin before and after plasma treatment: (**a**–**e**) lycopene, (**f**–**j**) capsanthin.

SEM-EDX analysis was performed to examine the morphology and elemental information of the aluminum samples at the microscale before and after plasma treatment, as shown in Figure 5. For the aluminum plate with lycopene, small particles that can be attributed to lycopene were seen on its surface. After plasma processing, the density of these particles was apparently reduced, and this can be explained by the degradation of lycopene under plasma impacts. The EDX spectra also support this finding, where the C/O ratio greatly decreased after plasma processing, from 0.82 to 0.30. As to the Al plate with capsanthin, its surface was relatively smooth, without the presence of particles. The lack of particles is because capsanthin has better solubility than lycopene, and capsanthin molecules were completely dissolved in the cyclohexane solution to get a uniform solution. Furthermore, no significant morphological changes were observed after plasma treatment. The elemental mapping shows C and O were uniformly distributed among the sample, with the C/O ratio drastically reduced from 9 to 1.5 after plasma treatment. The reduced content of C element further reveals the degradation of capsanthin by plasma.

**Figure 6:**
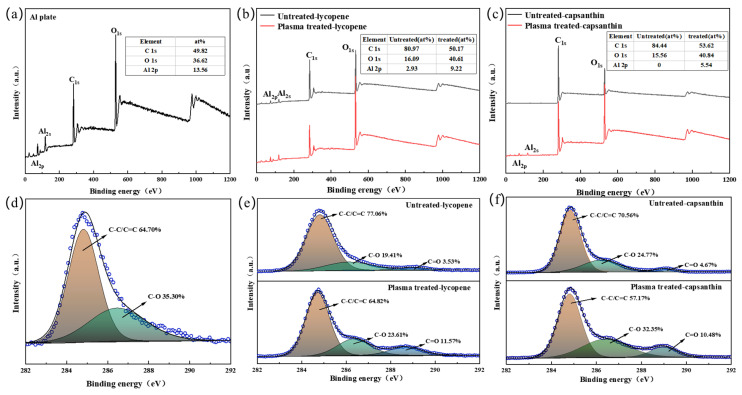
Overall XPS spectra of the samples before and after plasma treatment: (**a**) Al plate; (**b**) Al plates with lycopene; (**c**) Al plates with capsanthin; (**d**–**f**) high-resolution C 1s spectra; (**d**) Al plate; (**e**) Al plate with lycopene; (**f**) Al plate with capsanthin.

XPS characterization was conducted to examine the chemical composition and bonding configuration of the bare aluminum plate as well as the aluminum plates with the lycopene and capsanthin stains (Figure 6). All samples show the existence of C, O, and Al, as reflected by the peaks relating to C1s (285 eV), and O1s (532 eV). When the plates were covered by the stains, Al signals were considerably suppressed. However, after plasma processing, Al peaks became relatively strong, revealing the stains were degraded by plasma (Figure 6a–c). The deconvolution of the C1s band suggests the presence of C–C/C=C (64.7%) and C–O (35.3%) (Figure 6d). As to the aluminum plates covered by lycopene and capsanthin, the C–C/C=C (284.7 eV) content increased compared to the bare Al plate, which was above 70% for both samples (Figure 6e–f). The increased content of the carbon bonding configuration is because both lycopene and capsanthin are rich in the C element. Plasma processing led to a considerable decrease in the C–C/C=C content, which, in turn, suggests the degradation of lycopene and capsanthin. Furthermore, the relative amount of the C–O and C=O species increased, indicating the formation of carbon–oxygen groups under plasma impacts. As reported, plasma can promote the formation of carboxylate groups on the surfaces of materials, leading to a change in the surface wettability [11].

**Figure 7:**
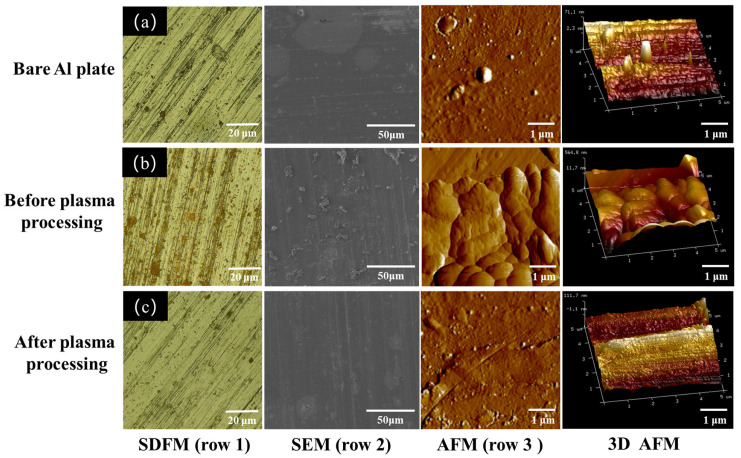
SDFM (row 1), SEM (row 2), and AFM images (rows 3, 4) of the different Al plates: (**a**) bare Al plate, (**b**) lycopene-covered Al plate before plasma processing, (**c**) Lycopene-covered Al plate after plasma processing.

The surface morphology of aluminum plates with lycopene before and after plasma treatment was first examined via a super depth-of-field microscope (row 1, Figure 7). All samples had apparent scratches on the surface at the microscale dimension. The Al plate with lycopene displayed the existence of rust red compounds on its surface, which was ascribed to the lycopene. However, after plasma processing, the lycopene was degraded, and no remarkable difference was observed between the plasma-treated Al plate and the bare Al plate. This phenomenon was also observed in SEM images, where irregular-shaped particles of lycopene exist on the surface of the Al plate but disappeared after plasma treatment (row 2). It can be inferred that lycopene stains can be rapidly degraded in the DBD plasma microreactor without damaging the metal plates [26,27]. AFM characterization was performed to further examine the topography of the samples before and after plasma treatment (row 3). The bare Al plate as well as the plasma-treated Al plate with lycopene displayed similar surface features, which were relatively flat and smooth compared to the lycopene-covered Al plate. The surface roughness was estimated to be 71.1 nm and 111.7 nm, respectively. However, for the sample without plasma processing, the surface was bumpy and exhibited grain morphology, with increased surface roughness observed (564.8 nm).

**Figure 8:**
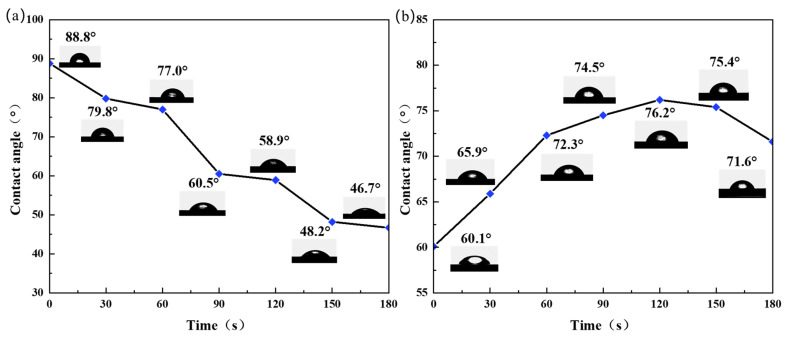
Water contact angles of aluminum plates covered by two types of stains with respect to plasma processing time: (**a**) lycopene, (**b**) capsanthin.

Figure 8 shows the water contact angles of the Al plates with lycopene and capsanthin treated with plasma at various conditions. A water contact angle of 88.8° was determined for the Al plate with lycopene before plasma treatment, which is very close to the value of the bare Al plate (80.0°). As the reaction time increased, the water contact angle of the Al plate gradually decreased, from 79.8° at 30 s to 46.7° at 180 s, indicating improved hydrophilicity (Figure 8a). For the Al plate with capsanthin, the water contact angle was measured to be 60.1°, suggesting its hydrophilic property [28]. However, when treated with plasma, the water contact angle initially increased with the processing time, from 65.9° at 30 s to 76.2° at 120 s. With further increases in the plasma processing time, the water contact angle decreased and reached 71.6° at 180 s (Figure 8b). The above phenomenon can be explained by the relatively large solubility of capsanthin in contrast to lycopene. As a result, fewer lycopene stains were absorbed on the surfaces of the Al plate than capsanthin, leading to reduced effects on the surface properties. Plasma treatment promotes hydroxylation on the surface of materials, rendering the surface more hydrophilic and increasing surface wettability. As to the capsanthin, due to its high solubility as well as the existence of hydrophilic groups like –OH and –COOH, etc., the water contact angle of the Al plate with capsanthin was smaller than the bare Al plate. However, after plasma treatment, capsanthin molecules were dissociated, resulting in an increased water contact angle. Once capsanthin molecules were dissociated, the hydroxylation reaction played a dominant role. Hydroxyl groups were formed on the surface of the Al plates, leading to increased hydrophilicity and a reduced water contact angle.

**Figure 9:**
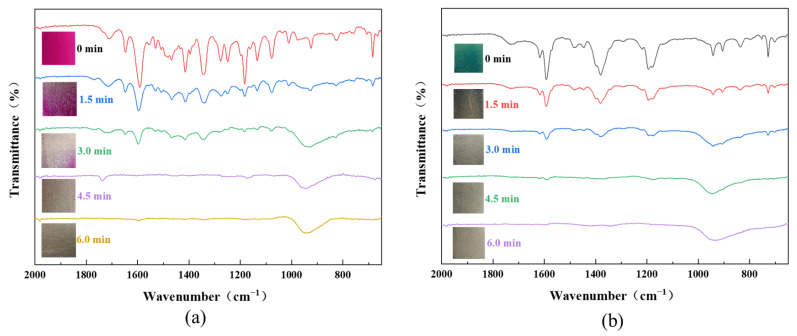
FT-IR absorption spectra of Al plates with different stains before and after plasma treatment for diverse times: (**a**) rhodamine B (RhB) and (**b**) malachite green (MG).

To evaluate the versatility of the DBD plasma technique for the cleaning of contaminants from metal surfaces, the method was extended to the degradation of stains like rhodamine B (RhB) and malachite green (MG). All experiments were performed at a plasma discharge power of 11.3 W, with an Ar/O_2_ flow rate of 48/2 sccm. Figure 9a displays the FT-IR spectra of the Al plates with RhB treated with plasma for different times. Before plasma processing, characteristic absorption bands of the RhB molecules were observed, including C=O stretching at 1717 cm^−1^, C=N stretching at 1642 cm^−1^, benzene ring vibration at 1595 cm^−1^, C–H bending at 1336 cm^−1^, and C–N stretching at 1181 cm^−1^ [29]. The intensity of these absorption bands apparently decreased after plasma treatment and disappeared when the processing time went beyond 4.5 min. Such an observation reveals the degradation of the RhB molecules. The FT-IR spectra of the Al plates with MG exhibited similar spectral features, where characteristic absorption bands of MG were seen for the Al plate with RhB before plasma processing. Specifically, the absorption bands at 1615 and 1586 cm^−1^ correspond to C=C aromatic stretching, the band at 1373 cm^−1^ is attributed to C–C stretching, and the band at 1172 cm^−1^ is due to C–N stretching [30]. With a prolonged plasma processing time, the intensity of these bands gradually decreased and almost disappeared with the processing time above 3 min. The results suggest the DBD plasma has universal application in the degradation of stains from the surface of metals.

**Figure 10:**
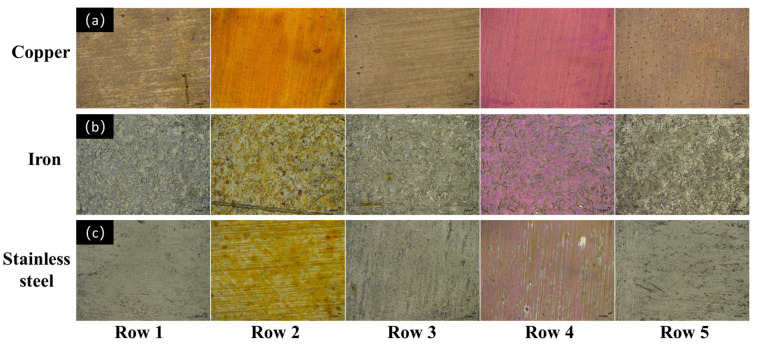
Digital microscope images of the bare metal plates as well as metal plates with different stains before and after plasma treatment: (**a**) copper plate, (**b**) iron plate, (**c**) stainless steel plate; row 1: metal plates without stains, row 2: metal plates with capsanthin, row 3: metal plates with capsanthin after plasma treatment, row 4: metal plates with RhB, row 5: metal plates with RhB after plasma treatment.

In addition to the stains, the universality of the DBD plasma cleaning method was also examined against different metals, including a copper plate, an iron plate, and a stainless-steel plate. All experiments were carried out at a plasma power of 11.3 W, with a plasma processing time of 3 min and an Ar/O_2_ flow rate of 48/2 sccm. Figure 10 provides typical optical images of the samples before and after plasma treatment, with capsanthin and rhodamine B as the stains. One can see the introduction of capsanthin will bring yellowish residues on the surface of metals. However, after plasma treatment for 3 min, these yellowish stains disappeared. No color difference was discerned between the plasma-treated metal samples and the bare metal plates, indicating the effective removal of capsanthin. As to the metal plates with rhodamine B, the color of the surface changed to purple, which is assigned to the color of RhB. However, after plasma processing, the purple color disappeared, suggesting the removal of the stains by plasma. Therefore, the DBD plasma technique has been confirmed to have high versatility in cleaning stains from different metals.

**Figure 11:**
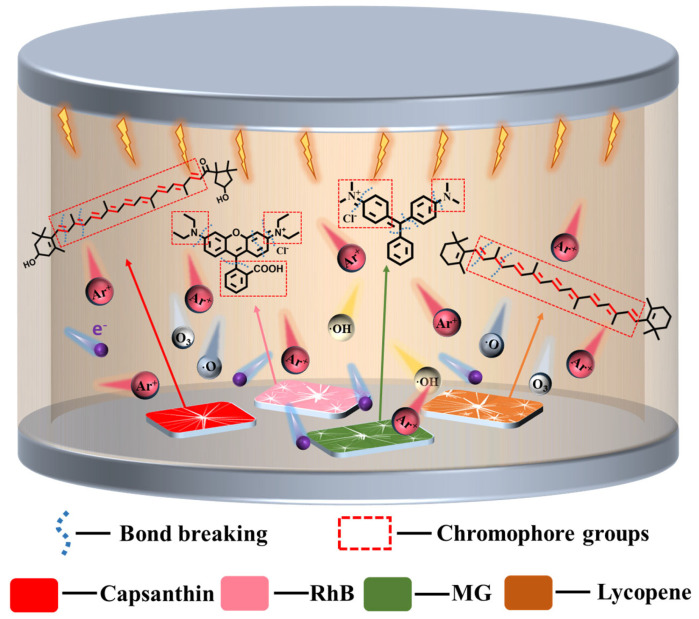
Schematic plasma degradation of stains on the surface of metals.

We have demonstrated an efficient and solvent-free way to clean stains from the surfaces of metals in a DBD plasma microreactor. Based on our experiment results, a schematic reaction process is presented in Figure 11. In plasma media, a series of reactive species exist, including electrons, Ar*, Ar^+^, and reactive oxygen species like O_2_^−^, OH·, O_2_*, and O_3_, etc. They can break the chemical bonds of the stains molecules to dissociate them effectively without damaging the metals. As reported, in microplasma, some electrons have energies exceeding 10 eV, which are larger than the chemical bond energies of the stains [14,31]. For instance, chemical bonds like C=C, C–C, C=O, C=C, C–C and C–N have bond energies in the range of 3–8 eV [32,33,34]. Thus, the chromophore groups can be easily dissociated by plasma. Meanwhile, due to the non-equilibrium feature of the DBD plasma, the gas temperature remains relatively low. Heat will not be accumulated during such a short processing time. Therefore, the bulk metals will not be damaged by plasma.

## 4. Conclusions

In this work, an efficient and solvent-free approach was demonstrated for the degradation of stains from metal surfaces using a DBD plasma microreactor. By selecting capsanthin as a study model, the influence of operational parameters on the decolorization efficiency of the plasma technique was investigated, including plasma discharge power, plasma processing time, and the O_2_ content in atmosphere. The results showed the increase in plasma power and processing time promoted the degradation of capsanthin stains. Furthermore, the addition of an appropriate amount of oxygen improved the decolorization efficiency, which was due to the formation of reactive oxygen species (ROS). Through systematic experiments, the optimum operation condition was determined to be the following: plasma power of 11.3 W, plasma processing time of 3 min, and Ar/O_2_ flow rate of 48/2 sccm.

Al plates with lycopene and capsanthin stains were treated in the DBD plasma microreactor at the optimum condition with the aim to study the evolution of functional groups, composition, morphology, bonding configuration, and wettability of the samples before and after plasma treatment. It has been revealed that both lycopene and capsanthin stains can be efficiently removed by DBD plasma without damaging the bulk metals. The universality of the DBD plasma approach was evaluated by extending the analytes to rhodamine B and malachite green, indicating they can be rapidly cleaned from the metal surfaces. Moreover, the types of metal plates were also researched using copper, iron, and stainless steel, suggesting the DBD plasma approach has good versatility in cleaning surface contaminates from different metals. Based on the above results, the degradation process of stains from metal surfaces in the DBD plasma microreactor was discussed. The demonstrated DBD plasma technique provides a simple, efficient, and solvent-free approach for the cleaning of stains from metal surfaces and is expected to be promising in industry cleaning.

## Data Availability

The data that support the findings of this study are available from the corresponding author upon reasonable request.

## References

[1] Gurău L., Coșereanu C., Timar M.C., Lungu A., Condoroţeanu C.D. (2022). Comparative Surface Quality of Maple (*Acer pseudoplatanus*) Cut through by CNC Routing and by CO_2_ Laser at Different Angles as Related to the Wood Grain. Coatings.

[2] Deng J., Zhao G., Lei J., Zhong L., Lei Z. (2022). Research Progress and Challenges in Laser-Controlled Cleaning of Aluminum Alloy Surfaces. Materials.

[3] Oya-Hasegawa M., Sato Y., Oya M. (2022). Analysis of interaction between mechanical force and chemical effect in cleaning phenomenon by probability density functional method. J. Surfactants Deterg..

[4] Filin S.A., Rogalin V.E., Kaplunov I.A. (2023). Methods of stabilization of halogenated hydrocarbons during automated physico-chemical cleaning of metal-optics. Procedia Struct. Integr..

[5] Bistis P., Cabedo P.A., Bakalis S., Groombridge M., Zhang Z.J., Fryer P.J. (2024). Mechanical cleaning of food soil from a solid surface: A tribological perspective. J. Food Eng..

[6] Lin L., Pho H.Q., Zong L., Li S., Pourali N., Rebrov E., Tran N.N., Ostriko K., Hessel V. (2021). Microfluidic plasmas: Novel technique for chemistry and chemical engineering. Chem. Eng. J..

[7] Vasikaran E.M., Murugesan P., Moses J.A., Anandharamakrishnan C. (2022). Performance of non-thermal plasma reactor for removal of organic and inorganic chemical residues in aqueous media. J. Electrost..

[8] Korner N., Beck E., Dommann A., Onda N., Ramm J. (1995). Hydrogen plasma chemical cleaning of metallic substrates and silicon wafers. Surf. Coat. Technol..

[9] Krüger P., Knes R., Friedrich J. (1999). Surface cleaning by plasma-enhanced desorption of contaminants (PEDC). Surf. Coat. Technol..

[10] Brandenburg R. (2017). Dielectric barrier discharges: Progress on plasma sources and on the understanding of regimes and single filaments. Plasma Sources Sci. Technol..

[11] Lin L., Rui L., Li C., Liu Q., Li S., Xia Y., Hu H., Yang W., Xu H. (2021). Study on CO_2_-based plasmas for surface modification of polytetrafluoroethylene and the wettability effects. J. CO_2_ Util..

[12] Cheng L., Ghobeira R., Cools P., Luthringer B., Asadian M., Geyter N.D., Liu Z., Yan K., Morent R. (2021). Comparing medium pressure dielectric barrier discharge (DBD) plasmas and classic methods of surface cleaning/activation of pure Mg for biomedical applications. Surf. Coat. Technol..

[13] Rodríguez-Villanueva C., Encinas N., Abenojar J., Martínez M.A. (2013). Assessment of atmospheric plasma treatment cleaning effect on steel surfaces. Surf. Coat. Technol..

[14] Mariotti D., Mohan Sankaran R. (2010). Microplasmas for nanomaterials synthesis. J. Phys. D Appl. Phys..

[15] Chiang W.-H., Mariotti D., Mohan Sankaran R., Gary Eden J., Ostrikov K. (2019). Microplasmas for Advanced Materials and Devices. Adv. Mater..

[16] Rui L., Li Z., Hu H., Liu Y., Liu Y., Starostin S., Hessel V., Lin L. (2023). Fluorescent Patterning of Polymeric Substrates with Rare-Earth-Doped Nanophosphors Assisted by Atmospheric Pressure Plasma. Ind. Eng. Chem. Res..

[17] Mao Z., Asai Y., Yamanoi A., Seki Y., Wiranata A., Minaminosono A. (2022). Fluidic rolling robot using voltage-driven oscillating liquid. Smart Mater. Struct..

[18] Mao Z., Yoshida K., Kim J.-W. (2019). A droplet-generator-on-a-chip actuated by ECF (electro-conjugate fluid) micropumps. Microfluid. Nanofluid..

[19] Wang R., Yang Y., Chen S., Jiang H., Martin P. (2021). Power Calculation of Pulse Power-Driven DBD Plasma. IEEE Trans. Plasma Sci..

[20] De Lima Petito N., da Silva Dias D., Costa V.G., Falcao D.Q., de Lima Araujo K.G. (2016). Increasing solubility of red bell pepper carotenoids by complexation with 2-hydroxypropyl-beta-cyclodextrin. Food Chem..

[21] Chen X., Magniez K., Zhang P., Kujawski W., Chen Z., Dumée L.F. (2023). A “Green” Stirring Plasma Functionalization Strategy for Controllable Oxygen-Containing Functional Groups on Octa-Methyl POSS Microstructure. Nanomaterials.

[22] Fang Z., Shao T., Wang R., Yang J., Zhang C. (2016). Influences of oxygen content on characteristics of atmospheric pressure dielectric barrier discharge in argon/oxygen mixtures. Eur. Phys. J. D.

[23] Baranski R., Baranska M., Schulz H. (2005). Changes in carotenoid content and distribution in living plant tissue can be observed and mapped in situ using NIR-FT-Raman spectroscopy. Planta.

[24] Baranska M., Schütze W., Schulz H. (2006). Determination of lycopene and beta-carotene content in tomato fruits and related products: Comparison of FT-Raman, ATR-IR, and NIR spectroscopy. Anal. Chem..

[25] Heriyanto, Gunawan I.A., Fujii R., Maoka T., Shioi Y., Kameubun K.M.B., Limantara L., Brotosudarmo T.H.P. (2021). Carotenoid composition in buah merah (Pandanus conoideus Lam.), an indigenous red fruit of the Papua Islands. J. Food Compos. Anal..

[26] Lin L., Rui L., Tao Y., Li Q., Chiang W.-H., Xu H. (2022). Surface modification of metal substrates using dielectric barrier discharge plasma and the wettability study. J. Taiwan Inst. Chem. Eng..

[27] Mao Z., Chen J., Li G., Wang D., Yuan Z., Fahlman B.D. (2016). Damage-Free Removal of Residual Carbon in a Dielectric Barrier Discharge (DBD) Plasma for Carbothermal-Synthesized Materials. Chem. Mater..

[28] Wei Z., Gu J., Ye Y., Fang M., Lang J., Yang D., Pan Z. (2020). Biodegradable poly (butylene succinate) nanofibrous membrane treated with oxygen plasma for superhydrophilicity. Surf. Coat. Technol..

[29] Fang Y., Zhou A., Yang W., Araya T., Huang Y., Zhao P., Johnson D., Wang J., Ren Z.J. (2018). Complex Formation via Hydrogen bonding between Rhodamine B and Montmorillonite in Aqueous Solution. Sci. Rep..

[30] Sartape A.S., Mandhare A.M., Jadhav V.V., Raut P.D., Anuse M.A., Kolekar S.S. (2017). Removal of malachite green dye from aqueous solution with adsorption technique using Limonia acidissima (wood apple) shell as low cost adsorbent. Arab. J. Chem..

[31] Lin L., Wang Q. (2015). Microplasma: A New Generation of Technology for Functional Nanomaterial Synthesis. Plasma Chem. Plasma Process..

[32] Horiuchi S., Tachibana Y., Yamashita M., Yamamoto K., Masai K., Takase K., Matsutani T., Kawamata S., Kurashige Y., Yanai T. (2015). Multinuclear metal-binding ability of a carotene. Nat. Commun..

[33] Carta C.L. (2014). The Effects of Medium on the UV-Induced Photodegradation of Rhodamine B Dye. Master’s Thesis.

[34] Zhao L., Zhi M., Frenking G. (2022). The strength of a chemical bond. Int. J. Quantum Chem..

